# Innovative Bioprocess Strategies Combining Physiological Control and Strain Engineering of *Pichia pastoris* to Improve Recombinant Protein Production

**DOI:** 10.3389/fbioe.2022.818434

**Published:** 2022-01-26

**Authors:** Arnau Gasset, Xavier Garcia-Ortega, Javier Garrigós-Martínez, Francisco Valero, José Luis Montesinos-Seguí

**Affiliations:** ^1^ Department of Chemical, Biological and Environmental Engineering, School of Engineering, Universitat Autònoma de Barcelona, Bellaterra, Spain; ^2^ QuBi Lab, Department of Biosciences, Faculty of Sciences and Technology, Universitat de Vic-Universitat Central de Catalunya, Vic, Spain

**Keywords:** GAP promoter, *Pichia pastoris (Komagataella phaffii)*, physiological control, transcriptional analysis, recombinant gene dosage, respiratory quotient, hypoxia

## Abstract

The combination of strain and bioprocess engineering strategies should be considered to obtain the highest levels of recombinant protein production (RPP) while assuring product quality and process reproducibility of heterologous products. In this work, two complementary approaches were investigated to improve bioprocess efficiency based on the yeast *P. pastoris*. Firstly, the performance of two *Candida rugosa* lipase 1 producer clones with different gene dosage under the regulation of the constitutive *P*_*GAP*_ were compared in chemostat cultures with different oxygen-limiting conditions. Secondly, hypoxic conditions in carbon-limited fed-batch cultures were applied by means of a physiological control based on the respiratory quotient (*RQ*). Stirring rate was selected to maintain *RQ* between 1.4 and 1.6, since it was found to be the most favorable in chemostat. As the major outcome, between 2-fold and 4-fold higher specific production rate (*q*_*P*_) values were observed when comparing multicopy clone (MCC) and single-copy clone (SCC), both in chemostat and fed-batch. Additionally, when applying oxygen limitation, between 1.5-fold and 3-fold higher *q*_*P*_ values were obtained compared with normoxic conditions. Thus, notable increases of up to 9-fold in the production rates were reached. Furthermore, transcriptional analysis of certain key genes related to RPP and central carbon metabolism were performed. Results seem to indicate the presence of a limitation in post-transcriptional protein processing steps and a possible transcription attenuation of the target gene in the strains with high gene dosage. The entire approach, including both strain and bioprocess engineering, represents a relevant novelty involving physiological control in *Pichia* cell factory and is of crucial interest in bioprocess optimization, boosting RPP, allowing bioproducts to be economically competitive in the market, and helping develop the bioeconomy.

## 1 Introduction

The need for recombinant proteins has increased exponentially over the last years, generating a billionaire market in which biotechnology plays a crucial role (Dewan, 2017). Most biopharmaceuticals, industrial enzymes, and metabolites are produced *via* biological systems, also named “cell factories.” In this context, bioprocess development combining strain and bioprocess engineering has emerged as a significant target for its optimization from an industrial point of view.

Among other workhorse cell factories, the yeast *Pichia pastoris* (renamed as *Komagataella phaffii*) (Kurtzman, 2005) has received increasing attention in the field of recombinant protein production (RPP). It has relevant advantages over other alternatives: the capacity to grow to high cell densities in defined medium, the ability to perform post-translational modifications, and to secrete the product into the culture broth. Furthermore, there are many tools available for its genetic modification, including Crispr/Cas9 (Weninger et al., 2016). All these advantageous features have been thoroughly described elsewhere (Çalık et al., 2015; Yang and Zhang, 2018). Due to these attractive characteristics, *P. pastoris* has already been used to produce more than 5,000 recombinant proteins, including biopharmaceuticals and industrial enzymes.

Traditionally, most of these processes in *P. pastoris* have been based on the use of the *AOX1* promoter (*P*_*AOX1*_), the strong and methanol-inducible promoter of Alcohol oxidase 1, which was the first to be reported. Alternatively, the constitutive glyceraldehyde-3-phosphate dehydrogenase promoter *P*_*GAP*_ has also been widely selected for RPP over the last two decades, since it avoids the use of methanol, thus reducing the oxygen and heat removal requirements compared to methanol-based processes. Moreover, methanol transport and storage at an industrial scale is also considered a relevant drawback for large-scale processes (García-Ortega et al., 2019).

Regarding strain engineering of this yeast, different approaches have been applied with the objective of increasing the production and productivity of the new clone (Ahmad et al., 2014). The increase in heterologous gene dosage (Cámara et al., 2016; Sallada et al., 2019) and the overexpression of transcriptional factors (Jiao et al., 2018; Liu et al., 2020) are successful examples of how genetic tools can provide new strains with much improved RPP performance. Novel approaches, including both promoter engineering and discovery, have recently been reported to have a high impact on RPP (Prielhofer et al., 2013; Portela et al., 2018; Ergün et al., 2019; Garrigós-Martínez et al., 2021).

Concerning bioprocess optimization, the operational mode is a key factor in bioprocess design, batch or fed-batch being the most established modes for production at an industrial scale, while chemostat is more commonly used for strain characterization and bioprocess design (Looser et al., 2014; Kumar et al., 2020; Nieto-Taype et al., 2020a). Additionally, the application of different types of cell stress has been demonstrated to have a significant positive effect on RPP, so the established process strategies can be remarkably improved (García-Ortega et al., 2019; Chen et al., 2021). Some examples are the application of substrate starving periods (Garcia-Ortega et al., 2016; Çalık et al., 2018) and the implementation of oxygen limiting conditions (Baumann et al., 2008), which have shown a positive effect on RPP under *P*_*GAP*_ regulation.

Previous studies have shown that oxygen limitation in *P. pastoris* cultures growing over glucose as the sole carbon source causes an increase in metabolic fluxes throughout the glycolytic and ethanol generation pathways, as well as a reduction in TCA fluxes, which signifies a shift from respirative to respiro-fermentative metabolism (Baumann et al., 2010). It was reported that not only an increase in metabolic fluxes, but also an overexpression of certain genes was observed, both at the mRNA and protein level. One of the overexpressed genes was *TDH3* (glyceraldehyde-3-phosphate dehydrogenase), which has *P*_*GAP*_ as its natural promoter.

In later studies, it was demonstrated that different degrees of oxygen limitation have a positive effect on the production of an antibody fragment expressed under *P*_*GAP*_ regulation, increasing the specific production rate (*q*_*P*_) by up to 3-fold with respect to the non-limiting conditions (Garcia-Ortega et al., 2017). In that study, it was proposed that, in order to implement the desired oxygen-limiting conditions in different equipment and operational modes, a physiological and system-independent parameter should be used as a transferable operating criterion. Two physiological parameters, which are system-independent, were proposed with this aim: specific ethanol production rate (*q*_*EtOH*_) and respiratory quotient (*RQ*).

Regarding the heterologous product, lipases are considered promising biocatalysts for industrial applications (López-Fernández et al., 2020). Among them, *Candida rugosa* lipase (Crl) has been widely used by flavor, oil, and pharma industries so far (Ken Ugo et al., 2017; Vanleeuw et al., 2019). Specifically, seven isoenzymes of *C. rugosa* lipase (Crl1-7) have been identified, Crl1 being the most abundant in commercial lipase preparations (Ferrer et al., 2001).

In the present work, the combined effect of both strain engineering and bioprocess optimization strategies has been studied. Two distinct *P. pastoris* producer clones, with different gene copy number, producing Crl1 under *P*_*GAP*_ regulation have been tested to determine the effect of gene dosage on RPP. In addition, oxygen-limiting conditions have been applied to test its effect on lipase production. Initially, both clones were characterized in chemostat cultures to compare their performance and to assess the combined effect of the two aforementioned strategies. Subsequently, optimal conditions regarding oxygen limitation obtained in chemostat have been applied to fed-batch cultures with both clones using *RQ* as the controlled variable. Additionally, transcriptional analysis of key genes involved in cell metabolism and RPP were performed to identify the impact of oxygen limitation on gene expression regulation. The combination of both gene dosage and physiological control represents a relevant novelty to further improve the performance of *Pichia*-based RPP.

## 2 Materials and Methods

### 2.1 Clone Construction and Gene Dosage Determination

Two selected recombinant clones of *P. pastoris* with one and five copies of the *CRL1* gene under the regulation of the constitutive *GAP* promoter (*P*_*GAP*_) were used for this study. The clones were named as follows: Single-Copy Clone (SCC) and Multi-Copy Clone (MCC). By using the α-mating factor of *Saccharomyces cerevisiae* the recombinant protein is secreted into the culture broth. Both clones had already been used in a previous study, thus the clone construction and *CRL1* copy number determination were described previously (Nieto-Taype et al., 2020b).

### 2.2 Chemostat Cultivation

Biological duplicates of chemostat cultivations were performed in a 2 L *Biostat B* fermenter (Braun Biotech, Melsungen, Germany) with a working volume of 1 L at a dilution rate (*D*) of 0.10 h^−1^ (Garcia-Ortega et al., 2016). The batch medium contained per liter of distilled water: citric acid 1.8 g, glycerol 40 g, (NH_4_)_2_HPO_4_ 12.6 g, MgSO_4_·7H_2_O 0.5 g, KCl 0.9 g, CaCl_2_·2H_2_O 0.02 g, antifoam Glanapon 2000kz (Bussetti & Co., GmbH, Vienna, Austria) 0.05 ml, biotin 0.4 mg, and 4.6 ml PTM_1_ trace salts solution. The PTM_1_ solution contained per liter: CuSO_4_·5H_2_O 6.0 g, NaI 0.08 g, MnSO_4_·H_2_O 3.36 g, Na_2_MoO_4_·2H_2_O 0.2 g, H_3_BO_3_ 0.02 g, CoCl_2_ 0.82 g, ZnCl_2_ 20.0 g, FeSO_4_·7H_2_O 65.0 g, and 5.0 ml H_2_SO_4_ (95%–98%). The chemostat medium contained per liter of distilled water: citric acid 0.9 g, glucose 50 g, (NH_4_)_2_HPO_4_ 4.35 g, MgSO_4_·7H_2_O 0.65 g, KCl 1.7 g, CaCl_2_·2H_2_O 0.01 g, antifoam Glanapon 2000kz 0.2 ml, biotin 1.2 mg, and 15 ml PTM_1_ trace salts solution. In order to reduce the oxygen supply without altering the mixing conditions of the process, different mixtures of air and N_2_, maintaining a constant aeration flowrate of 0.8 vvm, were implemented. Temperature was kept at 25°C and pH was maintained at 6.0 with NH_4_OH 15% v/v. For each condition tested, samples were taken and analyzed at 3, 4, and 5 residence times (τ).

### 2.3 Fed-Batch Cultivation

Fed-batch cultures were performed in duplicates using a 5 L *Biostat B* fermenter (Sartorius Stedim, Goettingen, Germany). The specific growth rate (*μ*) was also fixed at 0.10 h^−1^ by applying an exponential pre-programmed feeding profile. The batch medium composition was the same as for chemostat cultivation. The fed-batch medium contained per liter of distilled water: glucose 400 g, MgSO_4_·7H_2_O 6.45 g, KCl 10 g, CaCl_2_·2H_2_O 0.35 g, antifoam Glanapon 2000kz 0.2 ml, biotin 0.2 mg, and 1.6 ml PTM_1_ trace salts solution. Again, temperature was also controlled at 25°C and pH at 6.0 with NH_4_OH 15% v/v. The procedure is described in detail elsewhere (Garcia-Ortega et al., 2013).

Target degree of oxygen limitation was reached by maintaining the *RQ* at the corresponding set-point value. To do so, the agitation was modified following heuristic rules: to increase *RQ*, agitation was reduced in order to decrease the oxygen transfer rate to the culture media and, therefore, decrease the oxygen uptake rate. To reduce *RQ*, the opposite action was performed.

### 2.4 Biomass Analysis and Consistency Check

Biomass concentration in terms of dry cell weight (DCW) was determined as described previously (Cos et al., 2005). The Relative Standard Deviation (RSD) was below 5%.

Biomass elemental composition was determined as described previously (Carnicer et al., 2009). For all chemostat and fed-batch fermentations, the carbon recovery data was above 90%. The experimental data was further verified using previously described standard data consistency and reconciliation procedures (Wang and Stephanopoulos, 1983; van der Heijden et al., 1994; Verheijen, 2009), applying the constraint that carbon and electron conservation relations were satisfied. In all cases, a 95% confidence level was reached in the statistical consistency test, so it was considered that no significant measurement errors were made.

### 2.5 Carbon Source and by-Product Quantification

Glycerol, glucose, as well as the potential fermentation by-products (ethanol, arabitol, and succinate) were determined *via* HPLC. The column and software used for this purpose are described elsewhere (Jordà et al., 2014). RSD was below 1%.

### 2.6 Inlet- and Off-Gas Analyses

A *BlueInOne FERM* gas analyzer (BlueSens, Herten, Germany) was used with both chemostat and fed-batch cultures. CO_2_ and O_2_ molar fractions and absolute humidity were monitored and recorded online from exhaust gas, and intermittently measured from inlet gas. The gas analyzer was re-calibrated every steady-state in chemostats and every fed-batch in order to assure an accurate measurement. The data obtained were used to calculate the respirometric parameters: oxygen uptake rate (*OUR*), carbon dioxide evolution rate (*CER*), their corresponding specific rates (*qO*_*2*_ and *qCO*_*2*_) and respiratory quotient (*RQ*). RSD was below 5%.

### 2.7 Lipolytic Activity Determination

Crl1 enzymatic activity was analyzed using a p-nitrophenyl butyrate (pNPB)-based assay as previously described (Garrigós-Martínez et al., 2019). One activity unit is defined as the amount of enzyme needed to obtain 1 µmol of product per min under assay conditions. RSD was below 1%.

### 2.8 Transcriptional Analysis

Samples of 1 ml were taken from both chemostat and fed-batch cultures, mixed with 0.5 ml of a 95% ethanol:5% phenol mixture and centrifuged at maximum speed for 2 min at 4°C. Samples were then stored at −80°C until RNA extraction.

For RNA extraction, samples were first mixed with 300 μL of glass beads (425–600 μm, Sigma-Aldrich, St. Louis, MO, United States) and vortexed with TissueLyser (Qiagen, Hilden, Germany). RNA extraction was then carried out with the SV Total RNA Isolation System kit (Promega, Madison, WI, United States) following manufacturer’s instructions.

cDNA synthesis and determination of transcriptional levels were performed as reported previously (Garrigós-Martínez et al., 2019; Nieto-Taype et al., 2020b), with the only exception that in this case the β-actin gene (*ACT1*) was selected as the house-keeping gene, as in earlier transcriptional analyses under hypoxic conditions (Adelantado et al., 2017).

## 3 Results and Discussion

### 3.1 Characterization of Hypoxia and Gene Dosage Effects on Recombinant Crl1 Production and Cell Physiology Using Chemostat Cultures

#### 3.1.1 Effect of Hypoxia and Gene Dosage on Substrate Consumption and Crl1 Production

To evaluate the combined effect of gene dosage and oxygen limitation on *P. pastoris* physiology and Crl1 production, a set of chemostat cultures was performed using SCC and MCC and applying seven different oxygen supply conditions, ranging from 21% to 8% of oxygen in the inlet gas. A previous study established a dilution rate of *D = μ =* 0.10 h^−1^ to maximize *q*_*P*_ (Nieto-Taype et al., 2020b), therefore this *D = μ* was selected for testing all conditions.

**FIGURE 1:**
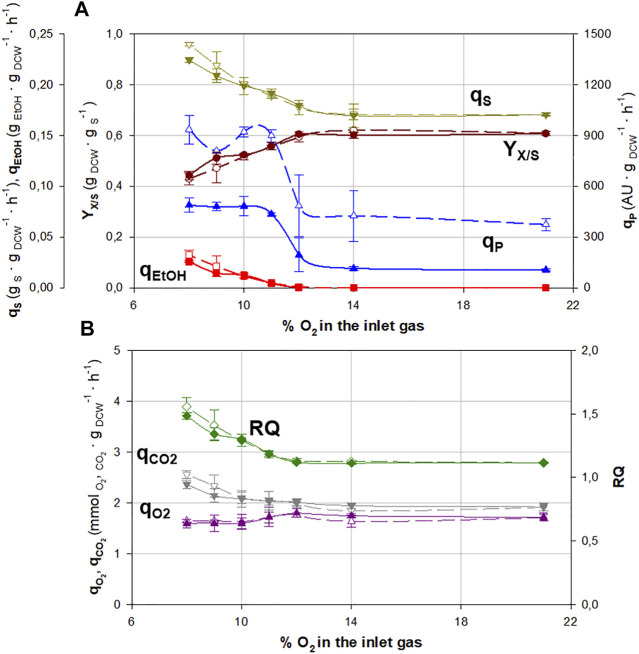
Main physiological and production parameters for continuous cultures at different molar fractions of oxygen in the inlet gas for single-copy clone (SCC, filled symbols) and multicopy clone (MCC, void symbols). **(A)** Specific substrate consumption rate, *q*_*S*_ (▼); overall biomass-to-substrate yield, *Y*_*X/S*_ (●); specific ethanol production rate, *q*_*EtOH*_ (∎); and specific Crl1 production rate, *q*_*P*_ (▲). **(B)** Respiratory quotient, *RQ* (◆); specific oxygen consumption rate, *qO*_*2*_ (▲); and specific carbon dioxide production rate, *qCO*_*2*_ (▼). Error bars represent the SD of biological replicates.

The variation of different key parameters (specific substrate consumption rate, *q*_*S*_; biomass-to-substrate yield, *Y*_*X/S*_; specific ethanol production rate, *q*_*EtOH*_; and *q*_*P*_) at different oxygen supply levels can be observed in Figure 1A for both SCC and MCC. The numeric values of these parameters are also shown in Supplementary Table S1.

Generally, the behavior of both clones can be seen to be rather similar, except for *q*_*P*_, so it can be stated that having a higher gene dosage does not significantly affect the yeast’s physiology except for Crl1 production.

In addition, the results show that the increase in *q*_*P*_ between both clones is not proportional to the increase in gene dosage, since MCC has between 2- and 3-fold higher *q*_*P*_ values than SCC while the gene copy number is 5 *versus* 1. Similar effects were also reported previously (Macauley-Patrick et al., 2005; Aw and Polizzi, 2013; Cámara et al., 2016).

Regarding oxygen limitation, no physiological changes are observed within the range of 21%–14% of oxygen in the inlet gas. As of 12% and lower, oxygen availability becomes a crucial factor. This implies that cells cannot completely oxidize the substrate, and so less energy is obtained from glucose, which causes a decrease in overall biomass-to-substrate yield (*Y*_*X/S*_). Then, since the same amount of substrate is consumed and less biomass is generated, the specific substrate consumption rate (*q*_*S*_) increases. This fact is confirmed by the absence of glucose in the culture medium.

At this point, around 12% of oxygen, there is a shift from respirative to respiro-fermentative metabolism (Baumann et al., 2010; Garcia-Ortega et al., 2017), confirmed by the accumulation of fermentative by-products such as ethanol, arabinitol, and succinic acid, which were already observed in similar glucose-based hypoxic cultures (Carnicer et al., 2009; Baumann et al., 2010; Adelantado et al., 2017; Garcia-Ortega et al., 2017). The specific ethanol production rate (*q*_*EtOH*_ = 0.0210 g_EtOH_·g_DCW_^−1^·h^−1^) is comparable to that observed in similar hypoxic chemostat cultures (*q*_*EtOH*_ = 0.0189 g_EtOH_·g_DCW_^−1^·h^−1^) with the same *D = μ* = 0.10 h^−1^, and the same hypoxic level (*RQ* = 1.4 ± 0.1) expressing a Fab antibody fragment (Adelantado et al., 2017). It has been described that high ethanol concentrations can result in yeast growth inhibition (Ergün et al., 2019; Wehbe et al., 2020). However, the concentrations reached in these fermentations did not attain these inhibitory levels.

As the oxygen molar fraction in the inlet gas decreases, hypoxia increases, but no glucose accumulation is observed until the oxygen molar fraction in the inlet gas drops below 8%, where bioreactor washout could be observed. This indicates that, under such conditions, critical dilution (*D*_*C*_) < 0.10 h^−1^ because *μ*_max_ decreases substantially.

Regarding Crl1 production, under hypoxic conditions around a 5-fold and 2.5-fold increase in *q*_*P*_ is observed for SCC and MCC, respectively, compared with normoxic conditions. Strikingly, it is worth mentioning that *q*_*P*_ reaches rather similar values under all normoxic conditions and under all hypoxic conditions, regardless of the oxygen limitation level. The highest variability in terms of *q*_*P*_ is observed in the transition between normoxic and hypoxic conditions (around 12% of oxygen molar fraction in the inlet gas) as previously described (Garcia-Ortega et al., 2017).

#### 3.1.2 Effect of Hypoxia and Gene Dosage on Respirometric Parameters

In Figure 1B, the variation in respirometric parameters (specific oxygen consumption rate, *qO*_*2*_; specific carbon dioxide evolution rate, *qCO*_*2*_; and *RQ*) can be observed. The value of these parameters under normoxic conditions is very similar to those reported in previous glucose-based cultures (Nieto-Taype et al., 2020b; Zavec et al., 2020). As observed in the previous section, both clones performed similarly, regardless of the gene dosage.

From 21% to 14% of oxygen in the inlet gas, the respirometric behavior of both clones is constant, corresponding to the normoxic state. When the oxygen molar fraction is reduced below 12%, the biomass has less oxygen available, so *qO*_*2*_ decreases and dissolved oxygen is around 0% (air saturation). This, combined with a slight increase in *qCO*_*2*_, has a direct impact on *RQ* values, which present a linear increase from 1.1 under normoxic conditions to 1.6 with the most severe hypoxic conditions, indicating a direct correlation between *RQ* and the degree of oxygen limitation.

Although the molar fraction of oxygen in the inlet gas is an easy-to-modify parameter, it cannot be generically correlated with hypoxia and the effect that it generates. The reason being that oxygen availability also relies on other factors: gas flow rate, stirring rate, temperature of the culture broth, and the configuration of the agitation/aeration system. On the other hand, *RQ* is a parameter that provides very valuable information regarding the degree of oxygen limitation that affects the biomass. Moreover, it does not depend on the culture system, but solely and exclusively on the physiological state of the cells. Thus, *RQ* can be used as a transferable operating criterion to apply the same hypoxic level to a different system.

*q*_*EtOH*_ could be also considered a reporting parameter of the hypoxic conditions, but its on-line determination would represent additional complexity because the on-line determination of biomass and ethanol concentrations would be necessary. In contrast, *RQ* control is expected to be less complex, being a parameter commonly determined on-line from off-gas analysis.

**FIGURE 2:**
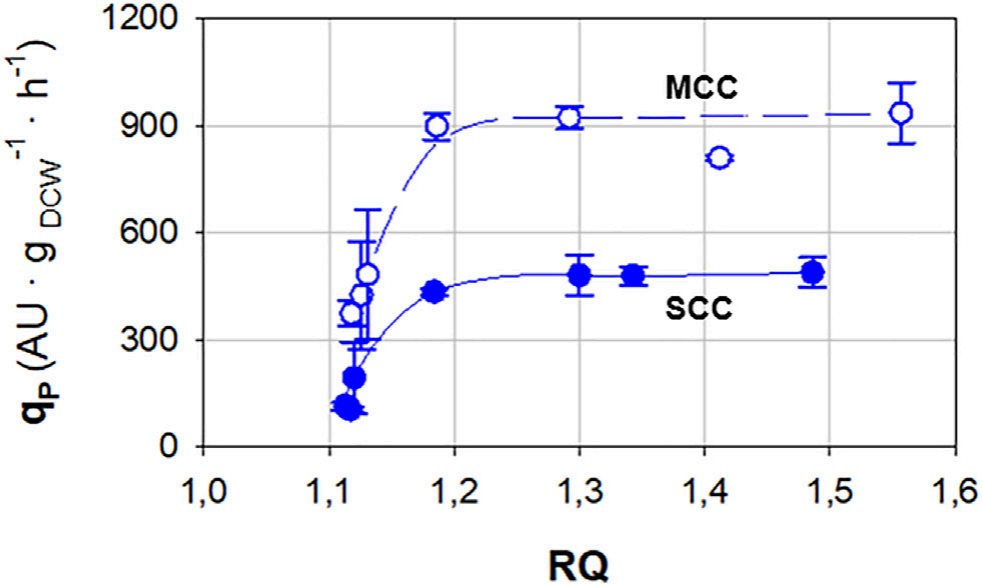
Relationship between specific Crl1 production rate (*q*_*P*_) and respiratory quotient (*RQ*) for single-copy clone (SCC, filled symbols) and multicopy clone (MCC, void symbols) obtained in chemostat cultures. Error bars represent the SD of biological replicates.

In Figure 2, the relationships between *q*_*P*_ and *RQ* for SCC and MCC are presented. In both cases, a set of grouped dots with low *q*_*P*_ and *RQ* values is observed, which correspond to normoxic conditions. When *RQ* reaches values around 1.2, a sudden rise in *q*_*P*_ can be observed, which becomes saturated when *RQ* > 1.3. From this point and above, *q*_*P*_ remains rather constant regardless of the *RQ* value.

Considering both strategies applied, namely gene dosage increase and oxygen limitation, an improvement of about one order of magnitude for *q*_*P*_ is achieved comparing SCC in normoxia (*q*_*P*_ = 106 Crl1 UA·g_DCW_^−1^·h^−1^) with MCC in hypoxia (*q*_*P*_ = 934 Crl1 UA·g_DCW_^−1^·h^−1^).

Once the combined effect of gene dosage and oxygen limitation was characterized, the next step was to transfer the bioprocess to a fed-batch mode, which represents a novel approach, as yet untested.

### 3.2 Fed-Batch Culture Strategy to Improve Bioprocess Efficiency

#### 3.2.1 Agitation Rate as Manipulated Variable to Achieve Target RQ

A set of fed-batch cultures were performed with a feeding strategy consisting of a pre-programmed exponential profile with *μ* = 0.10 h^−1^, mimicking the carbon-limited growth applied in chemostat.

Applying a constant level of oxygen limitation in a dynamic culture poses a challenge, since the control action over *RQ* will not be constant. If *RQ* is to be maintained at a certain value and biomass grows exponentially, oxygen supply to the culture should also be increased exponentially.

As observed in chemostat cultures, the variation of the oxygen fraction in the inlet gas is an easy-to-modify parameter by means of mixing nitrogen with air. However, at the industrial scale, the use of pure gases involves associated costs that should be avoided if possible. Instead, agitation is also an easy-to-modify parameter and is directly related to oxygen supply, since it notably affects the volumetric oxygen transfer coefficient (*k*_*L*_ *a*).

Thus, after having checked that the modification of both the oxygen molar fraction in the inlet gas and the stirring rate can be successfully used to maintain *RQ* within a particular range, it was decided to implement an *RQ* control based on agitation rate variation. This entails a new strategy, different from that implemented in chemostat cultures, which allows a saving in energy and pure gases, and, *a priori,* seems easier to implement in an industrial process, whenever proper mixing can be guaranteed and no heterogeneities in the bioreactor are produced.

The entire strategy applied in this work aims to be more robust and reproducible than that reported in previous studies, which was based on a start-and-stop substrate feeding that only targeted the avoidance of high ethanol accumulation, considered to be inhibitory, without taking into account other parameters than can affect *q*_*P*_ and productivity, such as *µ* and degree of oxygen limitation (Baumann et al., 2008).

As seen in Figure 2, the maximum *q*_*P*_ values were reached at *RQ* > 1.2. Even so, *RQ* values that are too high also cause an important ethanol production and a significant *Y*_*X/S*_ decrease. Consequently, in a discontinuous culture, ethanol accumulates in the culture broth and can reach inhibitory concentrations (Potvin et al., 2016; Ergün et al., 2019; Wehbe et al., 2020). On the other hand, a significant *Y*_*X/S*_ decrease entails a remarkably lower biomass production, since in a carbon-limited fed-batch, the open-loop feeding profile is designed considering a constant *Y*_*X/S*_. If *Y*_*X/S*_ decreases during the feeding phase due to a highly fermentative metabolism, so will *μ*. This, in turn, might cause a decrease in the specific production rate and productivity of the bioprocess.

A preliminary fed-batch, with *RQ* controlled above 1.8, was performed to check the possible ethanol inhibition effect. Ethanol accumulation achieved inhibitory concentrations (>30 g/L) and caused a progressive halt in biomass growth, as well as a *Y*_*X/S*_ reduction of more than 50% (data not shown). This ethanol inhibitory effect was not observed within the *RQ* range of 1.2–1.6. Consequently, an *RQ* set-point of 1.4 was selected. Thus, possible minor deviations of *RQ* due to the non-automatization of the *RQ* control are expected to have a smaller effect on *q*_*P*_, since it is quite constant within this *RQ* range, as observed in Figure 2.

#### 3.2.2 Crl1 Production in Fed-Batch Mode

**FIGURE 3:**
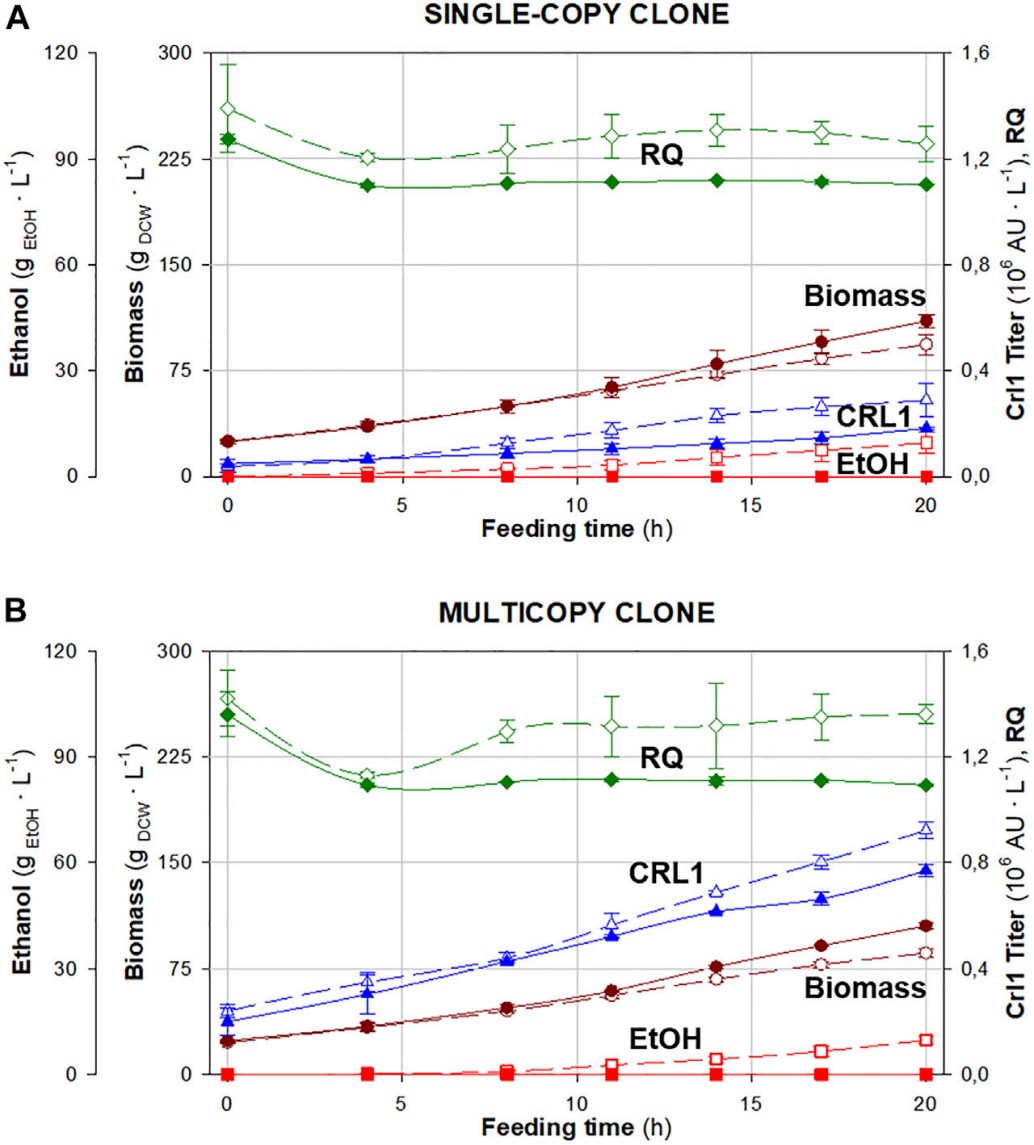
Time evolution of respiratory quotient (◆), biomass (●) and ethanol (∎) concentrations, and Crl1 activity (▲) in fed-batch cultures under different oxygen transfer conditions: normoxia (filled symbols) and hypoxia (void symbols). **(A)** Single-copy clone (SCC). **(B)** Multicopy clone (MCC). Error bars represent the SD of biological replicates.

The time evolution of biomass and ethanol concentrations, Crl1 titer, and *RQ* throughout the feeding phase, both under normoxic [dissolved oxygen higher than 30% (air saturation), *RQ* ≈ 1.1] and hypoxic conditions [dissolved oxygen ≈0% (air saturation), *RQ* set-point = 1.4] is shown in Figure 3. Accordingly, in Table 1, the averaged values of related key process parameters are presented.

**TABLE 1:** Averaged value of key process parameters obtained in fed-batch fermentations. Specific substrate consumption rate (*q*_*S*_), biomass-to-substrate yield (*Y*_*X/S*_), specific ethanol production rate (*q*_*EtOH*_), specific oxygen consumption rate (*qO*_*2*_), specific carbon dioxide evolution rate (*qCO*_*2*_), respiratory quotient (*RQ*) and specific Crl1 production rate (*q*_*P*_). ± indicate SD of the biological replicates.

	Single-copy clone		Multicopy clone	
O_2_ supply condition	Normoxia	Hypoxia	Normoxia	Hypoxia
*q*_*S*_ (g _S_ g _DCW_^−1^ h^−1^)	0.18 ± 0.01	0.21 ± 0.01	0.18 ± 0.01	0.22 ± 0.01
*Y*_*X/S*_ (g _DCW_·g _X_^−1^)	0.59 ± 0.01	0.47 ± 0.04	0.58 ± 0.01	0.50 ± 0.01
*q*_*EtOH*_ (g _EtOH_ g _DCW_^−1^ h^−1^)	n.d.	0.03 ± 0.01	n.d.	0.03 ± 0.01
*q*_*O2*_ (mmols _O2_ g _DCW_^−1^ h^−1^)	1.93 ± 0.07	1.67 ± 0.04	2.02 ± 0.04	1.78 ± 0.24
*q*_*CO2*_ (mmols _CO2_ g _DCW_^−1^ h^−1^)	2.13 ± 0.08	2.30 ± 0.10	2.23 ± 0.05	2.42 ± 0.19
*RQ*	1.11 ± 0.01	1.38 ± 0.09	1.10 ± 0.01	1.37 ± 0.07
*q*_*P*_ (AU g _DCW_^−1^ h^−1^)	164 ± 8	329 ± 27	696 ± 7	1,029 ± 45
*P·V* (AU)	(6.67 ± 0.45) × 10^5^	(1.07 ± 0.17) × 10^6^	(2.82 ± 0.10) × 10^6^	(3.62 ± 0.12) × 10^6^

As observed in chemostat cultures, both clones (SCC and MCC) exhibit similar behavior, except for Crl1 production. Biomass production decreases and Crl1 titers increase when applying hypoxia. Besides, *Y*_*X/S*_ decreases by about 20% and *q*_*S*_ increases accordingly compared with normoxic conditions. Ethanol accumulation up to 10 g/L is also detected under hypoxic conditions, although no growth inhibition is observed. Furthermore, arabitol (up to 7 g/L) and succinic acid (up to 1 g/L) are also detected in the culture broth.

It can be stated that *RQ* has been satisfactorily controlled within the desired range, being around 1.1 in normoxic fermentations and between 1.2 and 1.4 in hypoxic ones. However, small peaks in the *RQ* profile are detected, corresponding to manual control actions on the agitation rate carried out to regulate *RQ* (Supplementary Figure S1). As seen in Table 1, *qO*_*2*_ decreases by about 15% under hypoxic conditions, while *qCO*_*2*_ has the same value regardless of the oxygenation conditions. No significant differences were observed between both clones.

With regards to Crl1 production, *q*_*P*_ increases considerably with oxygen limitation. As observed in chemostat, the *q*_*P*_ increase for SCC (2-fold) is higher than for MCC (1.5-fold) when applying hypoxia.

From the results obtained in chemostat cultures, fed-batch data seem to indicate that, in this operational mode the application of oxygen limitation is less effective than in chemostat, since higher Crl1 titers were expected when implementing hypoxic conditions to fed-batch cultures, especially with MCC. This could be explained by the combination of some factors, among them: firstly, oxygen limitation causes metabolic stress, forcing cell metabolism towards the fermentative branch, generating a lower quantity of energetic molecules such as ATP (Carnicer et al., 2009; Baumann et al., 2010); secondly, cell aging caused by the time-effect related to fed-batch bioprocesses, which has also been described to affect RPP (Curvers et al., 2001; Cos et al., 2006), probably amplifies the metabolic stress, which does not occur in chemostats. Finally, saturation of secretion capacity has been also reported to be a possible bottleneck for high productive systems that could be limiting the expected Crl1 titers (Ahmad et al., 2014; Garcia-Ortega et al., 2016).

**FIGURE 4:**
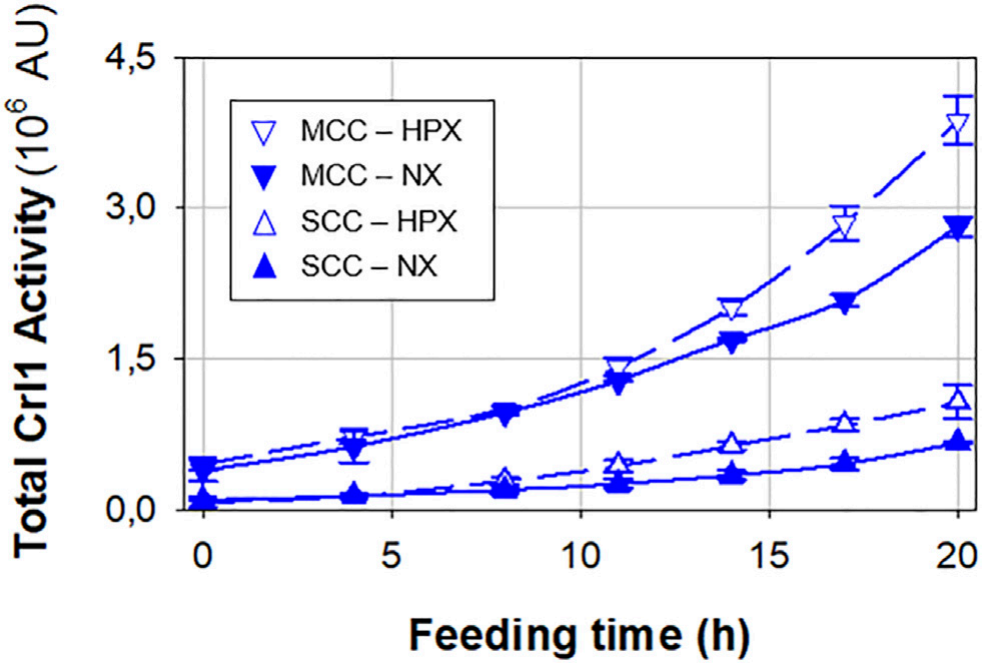
Total Crl1 activity for SCC (▲) and MCC (▼) obtained under normoxic (filled symbols) and hypoxic (void symbols) conditions. Error bars represent the SD of biological replicates.

Crl1 production is presented in Figure 4, where total Crl1 activity time profiles are plotted. SCC has a 70% higher Crl1 production under hypoxic rather than normoxic conditions, whereas the increase for MCC is only 20%. On the other hand, MCC presents an approximately 4-fold higher Crl1 production than SCC. This represents an overall 6.3-fold increase in terms of *q*_*P*_, considering both increased gene dosage and oxygen limitation, lower than that achieved in chemostat cultures.

### 3.3 Impact of Hypoxia and Gene Dosage at the Transcriptional Level

#### 3.3.1 Consistency Between Physiological Parameters and Gene Expression Levels

Transcriptional analysis of key genes was performed with the aim of identifying potential correlations between gene regulation and protein production under hypoxic conditions.

The genes selected were the recombinant *CRL1* gene, which allows the expression of the target product of the bioprocess, the glycolytic genes *TDH3* and *PGK1* that encode two crucial enzymes for central carbon metabolism, and the Unfolded Protein Response (UPR)-related genes *HAC1* and *KAR2*, described as an UPR-related transcription factor and an UPR-related chaperone, respectively (Guerfal et al., 2010; Raschmanová et al., 2019).

**FIGURE 5:**
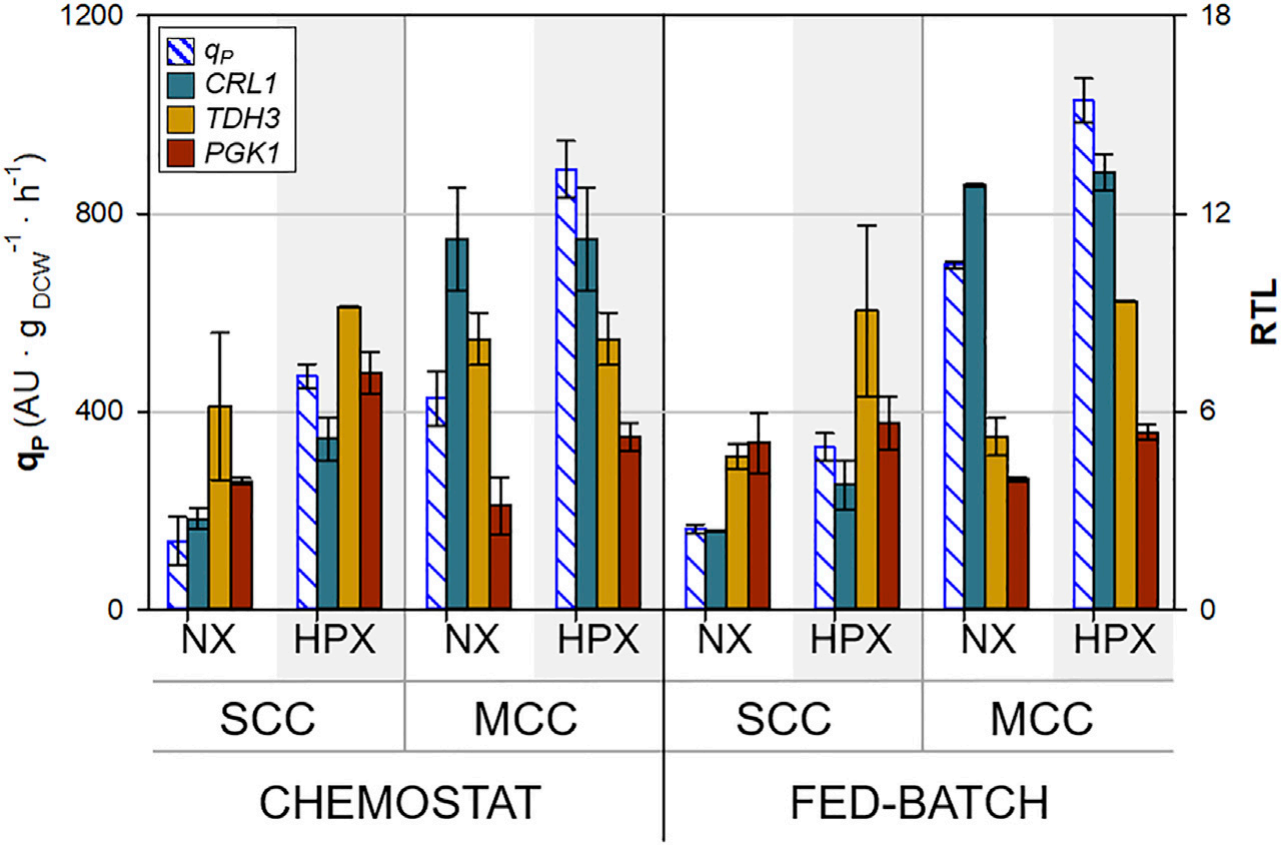
Comparison of *q*_*P*_ and relative transcript levels (RTL) of key genes under all conditions tested. Error bars represent the SD of all samples belonging to the same group of culture conditions (hypoxic and normoxic).

The transcription levels of the genes of interest have been studied in accordance with the protocol described in Section 2.8. The results of the relative transcription levels (RTL) for *CRL1, TDH3,* and *PGK1*, combined with the averaged *q*_*P*_ values, are shown *via* bar graphs in Figure 5. In order to facilitate a comprehensive comparison of both chemostat and fed-batch results, these are grouped into normoxic (1.1 < *RQ* < 1.2) and hypoxic results (*RQ* > 1.2) as the RTL of all the genes plotted presented no significant differences between any condition for each of the two groups (normoxic and hypoxic), as observed in Supplementary Table S2. No clear trends could be observed for *HAC1* and *KAR2* RTLs with respect to neither *RQ* nor *CRL1* gene dosage, so they are excluded from the graphs and the discussion (results presented in the Supplementary Table S2). Previous studies, in which the same two Crl1-producer clones were studied, showed that these genes remained unregulated under different specific growth rate (*µ*) conditions (Nieto-Taype et al., 2020b).

Regarding the glycolytic genes selected, *TDH3* and *PGK1*, as expected, there are no significant differences with respect to *CRL1* gene dosage in terms of transcript levels. Interestingly, the transcription levels of the glycolytic genes analyzed are boosted under oxygen limitation conditions. Accordingly, it could be stated that oxygen-limiting conditions have a significant effect on the upregulation of glycolysis-related gene expression. Additionally, in previous studies using the same clones but analyzing the effect of the specific growth rate, a direct correlation between *TDH3* RTL and *µ* was also observed. However, this effect was not that evident with *PGK1* RTL (Nieto-Taype et al., 2020b).

When comparing *q*_*P*_ and *CRL1* RTL, a similar trend could be observed: they increase in MCC with respect to SCC. In addition, oxygen-limiting conditions also increases *q*_*P*_ and *CRL1* RTL. Interestingly, it is clearly seen that the largest increase is due to the higher gene copy number rather than the effect of oxygen limitation.

It is also important to note that no clear differences could be observed between chemostat and fed-batch transcription results, although the effects of gene dosage and oxygen limitation seem to impact more on chemostat than on fed-batch cultures, except for the *TDH3* gene, where the differences between the normoxic and hypoxic state are clearer in fed-batch cultures.

#### 3.3.2 Effect of Gene Dosage on Gene Expression and Crl1 Production

**FIGURE 6:**
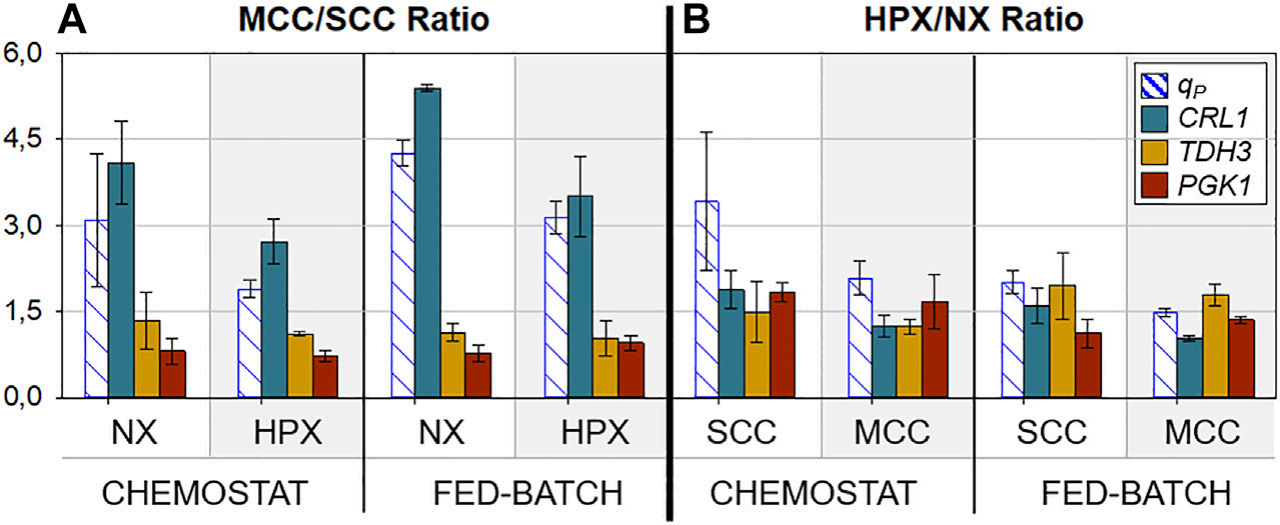
Ratios of *q*_*P*_ and relative transcript levels (RTL) of key genes under all conditions tested. **(A)** Ratio between MCC and SCC for each condition (hypoxic and normoxic). **(B)** Ratio between hypoxic conditions and normoxic conditions for each clone (SCC and MCC). Error bars calculated by error propagation of the corresponding quotients.

To provide further insights regarding the transcriptional results, two new parameters were defined: MCC/SCC Ratio, which corresponds to the RTL quotient of the aforementioned genes between multicopy and single-copy clones; and HPX/NX Ratio, which corresponds to the RTL quotient between hypoxic conditions and normoxic conditions. The former facilitates the comprehension of the effect of gene dosage on gene expression regulation throughout the different conditions tested, while the latter highlights the effect of oxygen limitation. The values of these ratios are plotted in Figure 6.

In relation to gene dosage (MCC/SCC Ratio), as expected, no effect on glycolytic reporting genes (*TDH3*, *PGK1*) is observed, since their gene regulation mechanisms remained unchanged. However, these results identified a possible bottleneck regarding Crl1 production in MCC, considering that the MCC/SCC *CRL1* RTL ratio is always slightly superior to the MCC/SCC *q*_*P*_ ratio, irrespective of the oxygenation conditions. This fact suggests a limitation in recombinant protein processing, including translation, folding, and/or secretion. Specifically, under normoxic conditions the MCC/SCC *CRL1* RTL ratio values are around 5, in consonance with the increased number of *CRL1* gene copies. On the other hand, MCC/SCC *q*_*P*_ ratio values are lower than 5. A similar effect at high *µ* with this Crl1 producer MCC was also reported and thoroughly discussed (Puxbaum et al., 2015; Nieto-Taype et al., 2020b).

Very interestingly, the values of the MCC/SCC *CRL1* RTL and MCC/SCC *q*_*P*_ ratios in chemostat with *D = µ* = 0.10 h^−1^ and under normoxic conditions are very similar to those obtained in the previous work, which demonstrates the robustness and accuracy of both the chemostat cultures and the transcriptional analysis (Nieto-Taype et al., 2020b).

According to results shown in Figure 5, both parameters studied in this work enhance the *CRL1* transcription rate. However, the gene dosage effect is reduced under hypoxic conditions, where both MCC/SCC *CRL1* RTL and *q*_*P*_ ratios are lower. This could indicate that, when combining both the heterologous gene copy number increase and hypoxic conditions, the transcriptional machinery is working closer to its upper rate limit, apart from the potential protein processing limitation mentioned previously. A plausible explanation for such an effect could be a finite availability of *TDH3*-related transcription factors, such as Gal4-*like* (*CRA1*), found to be crucial in the regulation of respiro-fermentative metabolism through the interaction with *P*_*GAP*_ and possibly other glycolytic promoters (Ata et al., 2017, 2018; Kalender and Çalık, 2020). Dramatically increasing the number of transcription factor binding sites, which occurs in MCC, with a limited pool of these transcription factors, could cause an attenuation of the transcription of all *P*_*GAP*_ regulated genes, as previously described for *P*_*AOX1*_ (Cámara et al., 2017; Garrigós-Martínez et al., 2019). However, in this work, no significant attenuation of either *TDH3* transcription or *q*_*S*_ was observed.

#### 3.3.3 Effect of Hypoxia on Gene Expression and Crl1 Production

Concerning the oxygen limitation effect (HPX/NX Ratio), Figure 6 shows that glycolytic genes are overexpressed by about 1.5-fold under hypoxic conditions, with small deviations. This is in consonance with the generally observed *q*_*S*_ increase of about 1.25-fold. On the other hand, regarding Crl1 production, and consistent with the above results, hypoxic conditions have less impact on MCC than on SCC, both at the transcription (*CRL1* RTL) and protein secretion (*q*_*P*_) levels.

To sum up, the combination of oxygen limitation and increased gene dosage has a synergic but not summatory effect at the transcriptional level. Accordingly, the increase in Crl1 production achieved by the combination of these two strategies is larger than that obtained using these two strategies separately. Moreover, the insights described shed light on the regulation of the expression of these genes, which could help identify bottlenecks in the RPP.

## 4 Conclusions

It can be concluded that both strain and bioprocess engineering can boost RPP. In this work, a 5-fold increase in gene dosage generated a remarkable increase in *q*_*P*_. However, the gene dosage increase does not present a linear correlation with the *q*_*P*_ increase, not being proportional. On the other hand, the application of cell stress has been shown to increase Crl1 production without having a significative impact on yeast growth. Overall, the application of oxygen limitation, specifically in the production of recombinant protein under *P*_*GAP*_ regulation under hypoxic conditions, combined with the increase in *CRL1* gene dosage produces a notable increase in *q*_*P*_—almost 9-fold higher—in chemostat cultures. When this novel strategy is transferred to a different operational mode, such as fed-batch cultivation, a slightly lower but also important increase in *q*_*P*_ is observed. This is the first time that the combinatorial effect of gene copy number and hypoxic conditions has been studied.

Considering the results of the transcription analyses carried out, the presence of a bottleneck downstream of the transcription steps has been observed in those clones with higher levels of recombinant production (MCC). This effect had already been observed with this MCC when growing at high *μ*, indicating a saturation of the protein production pathways. Moreover, it has also been proposed that there might be a transcription factor limitation in MCC under those conditions where protein production is maximized, since the *CRL1* RLT increase under hypoxic conditions is higher for SCC than for MCC.

Also, it has been demonstrated that both variable stirring rate and/or variable inlet gas composition can be used to implement oxygen limitation with the aim of carrying out a physiological control of the *P. pastoris* cell factory. Nevertheless, the first option newly applied could be a more feasible strategy in large scale mainly because of the energy saving and reduction of used extra gases.

In the present case, it can be stated that there is a wide *RQ* range that corresponds with a rather similar increase in *q*_*P*_. Consequently, manual heuristic control of *RQ* has been suitable for an exploratory study, but further work would be required to achieve an automated, accurate, and stable *RQ*-control. Accordingly, an adaptive proportional (P) or proportional-integral (PI) feedback *RQ*-controller with stirring rate as the manipulate variable could be implemented.

Finally, RQ has been proposed as a novel key physiological parameter to be used as a criterion in the scale-up of *P. pastoris* bioprocesses. It allows working under comparable oxygen availability conditions in different fermenters with distinct oxygen transfer efficiencies, and even in different operating modes. Moreover, an enhanced RPP bioprocess for other proteins of interest, whose transcriptional regulation is also oxygen-modulated, could be achieved.

## Data Availability

The original contributions presented in the study are included in the article/Supplementary Material, further inquiries can be directed to the corresponding author.
