# Supplementary material for: Innovative Bioprocess Strategies Combining Physiological Control and Strain Engineering of Pichia pastoris to Improve Recombinant Protein Production
Source: Front Bioeng Biotechnol. 2022 Jan 26;10:818434. doi: 10.3389/fbioe.2022.818434 (PMC8826567; doi:10.3389/fbioe.2022.818434)
Supplement: Supplementary file 1 [file DataSheet1.pdf]

TABLE S1: Value of key process parameters obtained in chemostat fermentations. Specific substrate consumption rate (q_S), biomass-to-substrate yield ($Y_{X/S}$), specific ethanol production rate (q_{EtOH}), specific oxygen consumption rate (qO_2), specific carbon dioxide evolution rate (qCO_2), respiratory quotient (*RQ*) and specific Crl1 production rate (q_P). ± indicate standard deviation (SD) of the biological replicates.

TABLE S1:

	**SINGLE-COPY CLONE**						
**% O_2 molar composition** in the inlet gas	**21**	**14**	**12**	**11**	**10**	**9**	**8**
q_S ($g_S \cdot g_{DCW}^{-1} \cdot h^{-1}$)	0.17 ± 0.01	0.17 ± 0.01	0.18 ± 0.01	0.19 ± 0.01	0.20 ± 0.01	0.21 ± 0.01	0.22 ± 0.01
$Y_{X/S}$ ($g_{DCW} \cdot g_X^{-1}$)	0.61 ± 0.01	0.60 ± 0.01	0.61 ± 0.008	0.56 ± 0.01	0.52 ± 0.01	0.51 ± 0.03	0.44 ± 0.02
q_{EtOH} ($g_{EtOH} \cdot g_{DCW}^{-1} \cdot h^{-1}$)	n.d.	n.d.	0.001 ± 0.001	0.004 ± 0.001	0.013 ± 0.003	0.015 ± 0.001	0.026 ± 0.001
q_{O2} (mmols $_{O2} \cdot g_{DCW}^{-1} \cdot h^{-1}$)	1.72 ± 0.08	1.75 ± 0.04	1.80 ± 0.08	1.71 ± 0.11	1.60 ± 0.11	1.60 ± 0.16	1.59 ± 0.08
q_{CO2} (mmols $_{CO2} \cdot g_{DCW}^{-1} \cdot h^{-1}$)	1.92 ± 0.07	1.94 ± 0.05	2.02 ± 0.07	2.03 ± 0.07	2.08 ± 0.16	2.14 ± 0.14	2.37 ± 0.08
RQ	1.12 ± 0.01	1.11 ± 0.01	1.12 ± 0.01	1.18 ± 0.03	1.30 ± 0.01	1.34 ± 0.05	1.49 ± 0.03
q_P ($UA \cdot g_{DCW}^{-1} \cdot h^{-1}$)	106 ± 6	115 ± 11	194 ± 100	435 ± 9	482 ± 56	480 ± 25	489 ± 42

	**MULTICOPY CLONE**						
**% O_2 molar composition** in the inlet gas	**21**	**14**	**12**	**11**	**10**	**9**	**8**
q_S ($g_S \cdot g_{DCW}^{-1} \cdot h^{-1}$)	0.17 ± 0.01	0.17 ± 0.01	0.18 ± 0.01	0.19 ± 0.01	0.20 ± 0.01	0.22 ± 0.01	0.24 ± 0.01
$Y_{X/S}$ ($g_{DCW} \cdot g_X^{-1}$)	0.61 ± 0.01	0.62 ± 0.01	0.59 ± 0.02	0.56 ± 0.01	0.52 ± 0.01	0.47 ± 0.06	0.43 ± 0.02
q_{EtOH} ($g_{EtOH} \cdot g_{DCW}^{-1} \cdot h^{-1}$)	n.d.	n.d.	0.001 ± 0.001	0.005 ± 0.001	0.012 ± 0.002	0.021 ± 0.009	0.032 ± 0.005
q_{O2} (mmols $_{O2} \cdot g_{DCW}^{-1} \cdot h^{-1}$)	1.71 ± 0.07	1.63 ± 0.12	1.76 ± 0.04	1.72 ± 0.19	1.63 ± 0.15	1.65 ± 0.02	1.64 ± 0.03
q_{CO2} (mmols $_{CO2} \cdot g_{DCW}^{-1} \cdot h^{-1}$)	1.92 ± 0.07	1.84 ± 0.12	1.99 ± 0.08	2.04 ± 0.19	2.10 ± 0.11	2.33 ± 0.22	2.56 ± 0.07
RQ	1.12 ± 0.01	1.13 ± 0.01	1.13 ± 0.02	1.19 ± 0.02	1.29 ± 0.05	1.41 ± 0.12	1.55 ± 0.07
q_P ($UA \cdot g_{DCW}^{-1} \cdot h^{-1}$)	374 ± 36	425 ± 150	483 ± 181	897 ± 37	920 ± 31	808 ± 6	934 ± 85

TABLE S2: Value of Relative Transcript Levels (RTL) of key genes: *CRL1*, *TDH3*, *PGK1*, *HAC1* and *KAR2*. ± indicate standard deviation (SD) of the biological replicates.

TABLE S2:

	**SINGLE-COPY CLONE**					
	**CHEMOSTAT**				**FED-BATCH**	
**% O_2 condition**	**21%**	**12%**	**10%**	**8%**	**NORMOXIA**	**HYPOXIA**
CRL1 RTL	2.53 ± 0.19	2.96 ± 0.51	4.71 ± 1.55	5.64 ± 0.63	2.39 ± 0.02	3.78 ± 0.73
TDH3 RTL	4.59 ± 0.35	7.75 ± 3.08	9.16 ± 2.44	9.19 ± 0.56	4.64 ± 0.38	9.06 ± 2.59
PGK1 RTL	3.83 ± 0.87	3.96 ± 0.39	6.72 ± 1.74	7.63 ± 0.64	5.04 ± 0.89	5.67 ± 0.80
HAC1 RTL	0.37 ± 0.20	0.22 ± 0.02	0.28 ± 0.02	0.25 ± 0.11	0.34 ± 0.13	0.54 ± 0.08
KAR2 RTL	0.67 ± 0.18	1.15 ± 0.68	0.69 ± 0.12	0.69 ± 0.05	0.59 ± 0.17	0.89 ± 0.01
	**MULTICOPY CLONE**					
	**CHEMOSTAT**				**FED-BATCH**	
**% O_2 condition**	**21%**	**12%**	**10%**	**8%**	**NORMOXIA**	**HYPOXIA**
CRL1 RTL	10.13 ± 0.49	12.34 ± 0.53	13.39 ± 2.90	14.75 ± 1.06	12.87 ± 0.05	13.25 ± 0.55
TDH3 RTL	7.65 ± 0.20	8.76 ± 1.84	9.97 ± 1.04	10.45 ± 0.10	5.24 ± 0.57	9.35 ± 0.02
PGK1 RTL	2.54 ± 0.06	3.75 ± 1.18	4.95 ± 0.72	5.54 ± 0.99	3.95 ± 0.06	5.36 ± 0.24
HAC1 RTL	0.20 ± 0.01	0.25 ± 0.15	0.25 ± 0.18	0.18 ± 0.11	1.21 ± 0.08	0.24 ± 0.02
KAR2 RTL	0.92 ± 0.27	0.89 ± 0.28	1.69 ± 0.28	1.27 ± 0.29	1.30 ± 0.11	0.80 ± 0.19

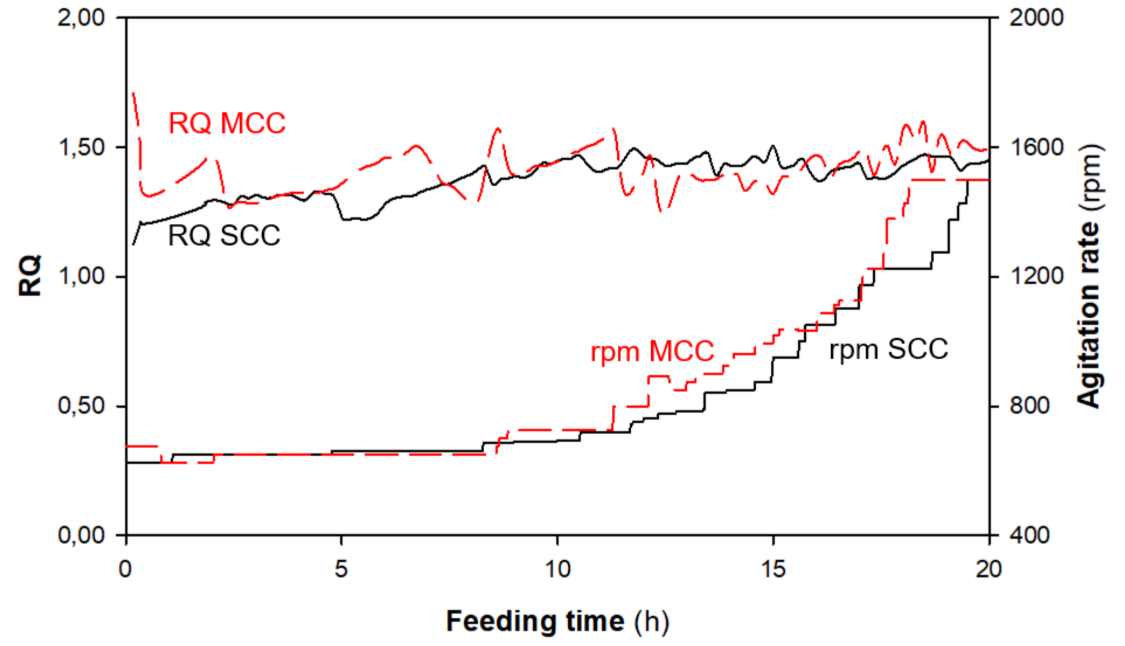

FIGURE S1: On-line time evolution of respiratory quotient (upper lines) and agitation rate (lower lines) for hypoxic fed-batch cultivations. Black and continuous lines for single-copy clone and red dashed lines for multicopy clone.
